# Novel application for the JAK inhibitor baricitinib in the treatment of Vogt–Koyanagi–Harada disease: a prospective cohort study

**DOI:** 10.3389/fimmu.2026.1736522

**Published:** 2026-03-17

**Authors:** Huan Luo, Bo Chen, Xian Zhang

**Affiliations:** 1 Joint Shantou International Eye Center of Shantou University and the Chinese University of Hong Kong, Shantou, Guangdong, China; 2 Shantou University Medical College, Shantou, Guangdong, China; 3 Department of Ophthalmology, Tongji Hospital, Tongji Medical College, Huazhong University of Science and Technology, Wuhan, Hubei, China

**Keywords:** baricitinib, JAK inhibitors, methlprednisolone, treatment, uveitis, Vogt-Koyanagi-Harada disease

## Abstract

**Purpose:**

The purpose of this study was to prospectively evaluate the clinical efficacy and safety of baricitinib, a selective Janus kinase (JAK)1/JAK2 inhibitor, in the treatment of patients with Vogt–Koyanagi–Harada (VKH) disease.

**Design:**

The study was designed as a prospective, open-label, longitudinal, single-center cohort study.

**Methods:**

We enrolled 38 patients (76 eyes) with VKH disease (including both initial-onset and recurrent/chronic cases) at Tongji Hospital, Wuhan, China, between 2022 and 2024. Patients were assigned to one of two treatment protocols based on disease severity and prior treatment history: group A received oral baricitinib (4 mg/day) combined with systemic methylprednisolone, and group B received oral baricitinib monotherapy. Clinical assessments were performed at baseline and at 1, 3, 6, 12, and 18 months. Primary outcome measures included best-corrected visual acuity (BCVA), anterior chamber (AC) cell and flare grades, subfoveal choroidal thickness (SFCT), subretinal fluid (SRF) height, and angiographic inflammatory scores (fluorescein angiography [FA]/indocyanine green angiography [ICGA]). Secondary outcomes included quality-of-life scores (25-item version of the Visual Function Questionnaire [VFQ-25], SF-36), adverse drug reactions (ADRs), and recurrence rates.

**Results:**

The cohort included 44 eyes with early-stage (initial-onset) and 32 eyes with late-stage (recurrent) VKH disease (mean age: 43.1 years ± 12.3 years; follow-up: 6–18 months). Inflammation control was achieved in all patients in both groups. In group A, the combination therapy led to rapid resolution of inflammation. In group B (monotherapy), significant improvements were observed from baseline to the final visit, including improved BCVA (0.12 to 0.68, *p* < 0.001), reduced AC cell grade (1.54 to 0.05, *p* = 0.007), decreased SFCT (537.6 to 252.4 µm, *p* = 0.007), and complete resolution of SRF (*p* < 0.001). FA and ICGA scores significantly decreased (*p* < 0.05), and VFQ-25 scores improved (82.6 to 92.9, *p* < 0.001). No severe ADRs or disease recurrences were observed during the follow-up period.

**Conclusion:**

This study demonstrates that baricitinib is a promising therapeutic option for both early- and late-stage VKH disease. It effectively controls choroidal inflammation, improves visual function, and allows for a substantial reduction in corticosteroid burden with a favorable safety profile.

**Clinical trial registration:**

http://www.chictr.org.cn, identifier ChiCTR2100048030.

## Introduction

Vogt–Koyanagi–Harada (VKH) disease is a multisystem autoimmune disorder characterized by a T-cell-mediated immune response against melanocytes in the uvea, inner ear, meninges, and integumentary system (1). In the eye, it manifests primarily as bilateral granulomatous panuveitis, with diffuse choroiditis and exudative retinal detachments being the hallmarks of the acute stage (2). VKH disease predominantly affects pigmented populations, including Asians, Hispanics, and Native Americans (3). In China, it accounts for a significant proportion of uveitis cases (approximately 13.5%) (4), and studies indicate that up to 38% of Chinese VKH patients present with visual acuity worse than 20/200 at their initial visit (5). Despite its prevalence, the precise etiology and pathogenesis of VKH disease remain incompletely understood. Without timely and effective intervention, this potentially blinding disease can severely compromise patients’ quality of life (1, 2).

According to revised diagnostic criteria (6), VKH disease encompasses two distinct clinical phases: the initial-onset acute uveitis stage and the chronic recurrent (or convalescent) stage (7, 8). Historically, earlier criteria often conflated the signs of these phases, leading to diagnostic ambiguity and treatment delays (9–11). Current treatment paradigms have evolved alongside these classification updates. While high-dose systemic corticosteroids remain the mainstay of therapy, there is increasing consensus on the early introduction of immunosuppressive agents in the acute phase to prevent chronicity (12–14). For the chronic recurrent phase, biological agents such as adalimumab (ADA) have emerged as effective options for refractory cases (15, 16). However, there remains a need for alternative therapies that offer both rapid inflammatory control and a favorable safety profile.

Baricitinib is an oral, selective Janus kinase (JAK)1 and JAK2 inhibitor, distinct from biologic agents, and is classified as a targeted synthetic small-molecule drug (17). It modulates cytokine signaling pathways by inhibiting the enzymatic activity of JAK1 and JAK2, thereby reducing the phosphorylation of signal transducers and activators of transcription (STATs). This blockade suppresses the activation and proliferation of key immune cells involved in autoimmune inflammation (18). While baricitinib was approved by the FDA in 2018 for rheumatoid arthritis (RA) (19), its application in noninfectious uveitis (NIU) is a rapidly evolving field (20, 21).

Recent investigations support the rationale for JAK inhibition in uveitis. For instance, Huang et al. utilized single-cell RNA sequencing to demonstrate the upregulation of the JAK1-mediated signaling pathway in patients with VKH and experimental autoimmune uveitis (EAU), identifying the JAK-STAT pathway as a critical therapeutic target (22). Furthermore, a cohort study by Vitale et al., which included patients with various noninfectious ocular inflammatory conditions treated with JAK inhibitors (including baricitinib, tofacitinib, and upadacitinib), confirmed that these agents are effective in controlling ocular inflammatory relapses (23). Despite these promising preliminary data, clinical evidence specifically focusing on baricitinib in VKH disease remains limited (21, 24).

In this prospective study, we investigated the efficacy and safety of a treatment regimen incorporating baricitinib, either as a monotherapy or in combination with corticosteroids, for the management of VKH disease. Over a follow-up period of 6 to 18 months, we evaluated clinical outcomes, assessed the drug’s effectiveness across both the early and late stages of the disease, and analyzed parameters associated with visual prognosis.

## Methods

### Study design

This observational prospective study was approved by the ethics committee of Tongji Hospital, Tongji Medical College, Huazhong University of Science and Technology, Wuhan, China, and was conducted from July 2022 to December 2024 following the ethical standards stated in the Declaration of Helsinki. Written informed consent was obtained from all the subjects. The study was registered at http://www.chictr.org.cn (registration number ChiCTR2100048030, accessed on 28 June 2021).

Firstly, VKH disease was diagnosed according to the classification criteria of the Standardization of Uveitis Nomenclature (SUN) study group 2021 (6), and eyes were classified as early- or late-stage of VKH disease according to clinical presentation and ophthalmologic examination, all of which were performed by an experienced ophthalmologist (XZ). Patients were subsequently divided into two groups, group A (oral baricitinib and methylprednisolone) and group B (oral baricitinib), according to their acceptance of steroid-related side effects and systemic conditions. Finally, the relevant treatment regimen was used for dosing and follow-up as follows: baricitinib at 4 mg/day, and after 3 months, the physician can adjust the dose of baricitinib according to the severity of the patient’s ocular inflammation by 1 mg/day every 3 months; methlprednisolone at 40 mg/day, decreased by 5 mg/day every 1 week. Patients were examined, and data were collected at baseline, 2 weeks, 1 month, 2 months, 3 months, 4 months, 5 months, and 6 months after enrollment, and all patients were followed up for at least 6 months. Adherence to oral baricitinib was monitored through patient diaries and pill counts at each monthly follow-up visit.

### Patient population

A total of 38 VKH disease subjects between 18 and 65 years of age were enrolled at the ophthalmology clinic of Tongji Hospital. The exclusion criteria were as follows: (i) Patients had no willingness to participate in the clinical trial; (ii) Diagnosis of infectious uveitis, such as viral uveitis, tuberculous uveitis, fungal infection, and *Toxoplasma gondii* infection, or could not be excluded; (iii) Complicated with severe glaucoma or cataract; (iv) Positive systemic screening for tuberculosis and hepatitis B without standardized antituberculosis and antihepatitis B virus treatment; (v) Combined with uncontrolled systemic diseases such as hypertension and diabetes; (vi) Hepatic or renal dysfunction; (vii) Pregnancy or lactation; and (viii) Previous severe cardiovascular and cerebrovascular diseases.

### History taking and ocular examinations

All the participants underwent comprehensive ophthalmic examinations, including refractive error assessment (AR-310A, Nidek, Gamagori, Japan), best-corrected visual acuity (BCVA), intraocular pressure (IOP) measurement (NT-510, Nidek, Gamagori, Japan), slit-lamp biomicroscopy, indirect ophthalmoscopy, color fundus photography (AFC-210, Nidek, Gamagori, Japan), scanning laser ophthalmoscopy (SLO) (Daytona [PT200], Optos, Dunfermline, Scotland), and spectral domain optical coherence tomography (SD-OCT) (Spectralis OCT, Heidelberg Engineering, Heidelberg, Germany). Fluorescence and indocyanine green angiography (Spectralis OCT, Heidelberg Engineering, Heidelberg, Germany) were performed when necessary to diagnose VKH disease.

Before administering baricitinib, a detailed prior medication history is obtained, and a comprehensive evaluation is performed, including PPD testing, chest radiography, screening for HIV, hepatitis B and C infection, syphilis screening, brain magnetic resonance imaging, cardiologic examination, complete blood count, and liver and kidney function tests. In order to monitor the side effects of baricitinib and methlprednisolone, liver and renal function, blood pressure, blood sugar levels, and routine blood test results were assessed every 2–4 weeks during treatment. All parameters and adverse drug reactions (ADRs) were recorded at the beginning and end of each visit. All surgical data were collected, especially for treatments that potentially improve visual function, such as cataract extraction.

### Acquisition of the primary observation indicators

#### Best corrected visual acuity

The BCVA was calculated using the Snellen chart and then converted to LogMar format. BCVA was transformed into a quantifiable visual acuity value in individuals with severely impaired visual function, such as “counting fingers/hand motion/light perception,” for statistical convenience (25).

#### Intraocular pressure

The IOP in both eyes of the patients was measured using a noncontact tonometer, and the values were recorded.

#### Anterior chamber cells and flare grade

Anterior chamber (AC) cell grade (0, 0.5+, 1+, 2+, 3+, 4+) and AC flare grade (0, 1+, 2+, 3+, 4+) were evaluated using the SUN working group grading scheme (26). Subsequently, these grade data were converted into measurement data for further statistical analyses. In the Nussenblatt scale, clarity of optic disc, retinal vessels, and nerve fiber layer is used to assess the vitritis into five grades (0, 1+, 2+, 3+, 4+) (27).

#### Subfoveal choroidal thickness and subretinal fluid height

A trained ophthalmic technician (JS) performed enhanced depth imaging OCT (EDI-OCT) scans from 9 a.m. to 11 a.m. Subfoveal choroidal thickness (SFCT) measurements were acquired using manual calipers from the retinal pigment epithelium/Bruch’s membrane (RPE/BM) reflective complex to the sclerochoroidal interface (28). If either of these interfaces could not be defined, even with improvement, the image was deemed unmeasurable. The subretinal fluid (SRF) height was measured from the tip of the RPE layer to the outside border of the detached retina at the fovea (29). The SRF thickness was measured if there was a hyporeflective layer corresponding to fibrinous membrane inside the exudative retinal detachment content covering the inner face of the RPE or the outer face of the retina. All of these measurements were conducted using ImageJ software by an experienced retinal physician (HL).

#### Fluorescein angiography score and indocyanine green angiography score

Fluorescein angiography (FA) and indocyanine green angiography (ICGA) were first performed using a standard protocol described previously (30), which also involved image acquisition and quality checking by a trained ophthalmic technician (JS). For scoring, FA and ICGA images were reviewed and scored independently by two clinicians (XZ and HL) using a validated FA/ICGA grading scale (31). The points attributed to each characteristic FA finding, such as macular edema, optic disc hyperfluorescence, retinal vascular staining and leakage, capillary leakage, retinal capillary nonperfusion, neovascularization of the optic disc or elsewhere, pinpoint leaks, retinal staining, and subretinal pooling, were added up to obtain a total score with a maximum possible score of 40. Similarly, a maximum score of 20 was assigned to ICGA abnormalities such as choroidal vasculitis, early stromal artery hyperfluorescence, hypofluorescent dark dots (HDD) or regions, and hyperfluorescence of the optic disc. Any disparity between the two independent readers was resolved by averaging the scores.

25-Item version of the Visual Function Questionnaire score and 36-item Short-Form Health Survey Questionnaire score.

One of the authors (HL) administered the vision-related quality of life (VR-QoL) and health-related quality of life (HR-QoL) measuring instruments during a face-to-face interview. To decrease the influence of the clinical encounter on patient replies, questionnaires were completed before the eye examination. The 25-item version of the Visual Function Questionnaire (VFQ-25) and 36-item Short-Form Health Survey Questionnaire (SF-36) were used to evaluate VR-QoL and HR-QoL, respectively. The VFQ-25 consists of 25 questions that assess 12 dimensions (32). The SF-36 consists of 36 items that measure eight categories of health status (33). Scores range from 0 to 100 on both questionnaires, with higher scores indicating a higher quality of life (32, 33). The reliability and validity of the Chinese versions of the VFQ-25 and SF-36 have been demonstrated (34, 35).

### Statistical analysis

For continuous parameters, the mean (SD) or median (interquartile range [IQR]) was used to describe the data. For categorical parameters, numbers or percentages were used. The Wilcoxon rank-sum test and Chi-square test were used to compare the differences in age, gender, disease categorization, and follow-up time between groups A and B. Student’s *t*-test was applied to verify the statistical significance of various variables at different follow-up time points (at baseline, 2 months, and 6 months) between groups A and B, and between early- and late-stage group. A related-sample *t*-test was used to examine the differences between the variables at pretreatment, posttreatment 2 months, and posttreatment 6 months in the four groups. SPSS Statistics 26.0 software (SPSS Inc., Chicago, IL, USA) was used for statistical analysis. A *p*-value < 0.05 was considered statistically significant.

## Results

### Demographic and baseline characteristics

A total of 38 patients (76 eyes) with active VKH disease were enrolled, comprising 16 men and 22 women (ratio 1:1.38). The mean age at onset was 43.1 years (range: 24–65 years). Based on clinical staging, 44 eyes were classified as early-stage (initial-onset) VKH disease, and 32 eyes were classified as late-stage (recurrent/chronic) VKH disease. All patients maintained regular follow-up for a minimum of 6 months, with a mean follow-up duration of 10.2 months ± 5.8 months (range: 6–18 months). Detailed baseline characteristics are provided in **Supplementary Table 1**.

### Comparison of treatment efficacy: combination therapy (group A) vs. monotherapy (group B)

**Figure 1:**
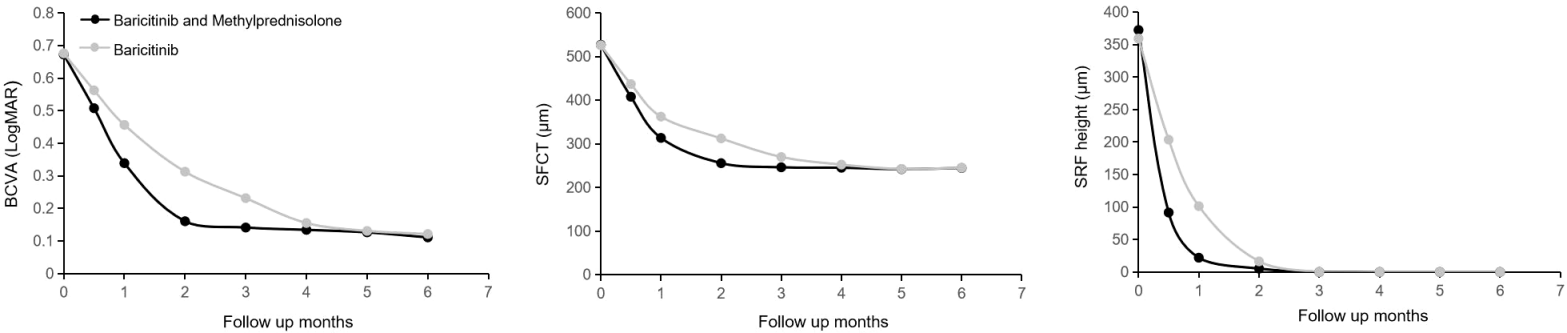
Changes in BCVA, SFCT, and SRF height during the follow-up period of all VKH disease patients, with oral baricitinib and methlprednisolone in Group A and oral baricitinib in Group B. BCVA, best-corrected visual acuity; LogMAR, logarithm of the minimum angle of resolution; SFCT, subfoveal choroidal thickness; SRF, subretinal fluid.

Baseline demographic and clinical characteristics showed no significant differences between patients treated with baricitinib plus methylprednisolone (group A) and those treated with baricitinib alone (group B) (**Tables 1**, **2**). Both treatment regimens achieved significant remission of inflammation. As shown in **Figure 1**, statistically significant improvements in BCVA, reductions in SFCT, and resolution of SRF height were observed in both groups over time.

**Table 1:** General demographic and clinical characteristics of VKH disease patients at various follow-up times.

Characteristics	Group A	Group B	*p*-value
**Number of patients**	21	17	
**Number of eyes**	42	34	
**Age (years)**	42 (31–57)	44 (30–58)	0.131^a^
Sex (*n*, %)			
**Female**	13 (61.90%)	9 (52.94%)	0.078^b^
**Male**	8 (39.10%)	8 (47.06%)	
Disease categorization (*n*, %)			
Early-stage	24 eyes (57.14%)	20 eyes (58.82%)	0.092^b^
Late-stage	18 eyes (42.86%)	14 eyes (41.18%)	
**follow-up (months)**	10 (7–12)	11 (8–13)	0.249^a^

Data are presented as mean ± SD (range) or median (*p*_25_–*p*_75_), unless otherwise indicated. *p*-values for differences in variables between groups A and B are based on the Wilcoxon rank-sum test, Chi-square test, or *t*-test as appropriate.

Bold values: *p < 0.05*.

a Wilcoxon rank-sum test.

b Chi-square test.

**Table 2:** Characteristics and comparison between the two groups of VKH disease eyes at various follow-up times.

Characteristics	Group A			Group B			*p*-value		
	At baseline	At 2 months	At 6 months	At baseline	At 2 months	At 6-months	*p*_1_-value^a^	*p*_2_-value^a^	*p*_3_-value^a^
BCVA (LogMAR)	0.66 ± 0.22	0.17 ± 0.08	0.11 ± 0.08	0.68 ± 0.28	0.31 ± 0.17	0.12 ± 0.09	0.713	< 0.001^*^	0.293
IOP (mmHg)	14.86 ± 2.54	16.55 ± 4.55	14.35 ± 2.43	14.13 ± 2.90	13.38 ± 2.16	14.76 ± 3.14	0.556	0.035^*^	0.606
AC cells grade (+)	1.55 ± 0.46	0.23 ± 0.23	0	1.54 ± 1.12	0.91 ± 0.46	0.05 ± 0.16	0.316	0.048^*^	0.821
AC flare grade (+)	1.61 ± 0.70	0.19 ± 0.37	0.03 ± 0.19	1.21 ± 0.95	0.52 ± 0.70	0	0.411	0.042^*^	0.971
SFCT (µm)	529.03 ± 114.01	275.11 ± 65.11	261.04 ± 63.06	537.55 ± 163.89	321.35 ± 109.46	252.44 ± 89.07	0.572	0.034^*^	0.768
SRF height (µm)	372.20 ± 34.25	5.12 ± 11.98	0	359.10 ± 78.61	16.32 ± 0.91	0	0.881	0.136	–
FA score	4.50 ± 1.20	0.54 ± 2.43	0	4.49 ± 1.69	1.47 ± 0.61	0.13 ± 0.35	0.708	0.438	0.930
ICGA score	10.73 ± 4.30	2.13 ± 4.85	0.75 ± 1.39	11.00 ± 2.83	4.09 ± 1.71	0.50 ± 1.07	0.921	0.024^*^	0.891
Total VFQ-25 score	82.00 ± 6.70	91.00 ± 6.09	90.10 ± 1.13	82.58 ± 9.27	93.88 ± 4.45	92.88 ± 3.73	0.976	0.216	0.962
Total SF-36 score	88.06 ± 18.52	92.38 ± 7.98	93.01 ± 6.01	89.58 ± 4.06	93.50 ± 5.37	93.91 ± 1.39	0.881	0.321	0.798

Data are presented as mean ± SD (range) or median (*p*_25_–*p*_75_), unless otherwise indicated. *p*-values for differences in variables between groups A and B are based on the Wilcoxon rank-sum test, Chi-square test, or *t*-test as appropriate. *p*_1_-, *p*_2_-, and *p*_3_-values indicate the differences between the two groups at baseline, 2 months, and 6 months, respectively.

*BCVA*, best-corrected visual acuity; *LogMAR*, logarithm of the minimum angle of resolution; *IOP*, intraocular pressure; *AC*, anterior chamber; *SFCT*, subfoveal choroidal thickness; *SRF*, subretinal fluid; *FA*, fluorescein angiography; *ICGA*, indocyanine green angiography; *VFQ-25*, 25-item version of the Visual Function Questionnaire; *SF-36*, 36-item Short-Form Health Survey Questionnaire.

^*^p < 0.05.

a *t*-test.

Notably, the rate of improvement differed between the two groups during the early phase. At the 2-month follow-up, group A (combination therapy) exhibited significantly greater reduction in SFCT and better BCVA compared to group B (**Figure 1**, *p* < 0.05). However, by the 6-month endpoint, these differences converged, and there were no statistically significant differences in BCVA, SFCT, or SRF height between the two groups. Similarly, scores on the VFQ-25 and SF-36 showed comparable improvements in quality of life at the final visit for both groups. Regarding safety and complications, no significant cataract progression was observed in either group during the 6-month follow-up period, and no patients required cataract surgery during the active treatment phase.

### Comparison of clinical outcomes: early- vs. late-stage VKH disease

VKH patients were divided into early- and late-stage groups in **Table 3**, and we summarized the characteristics of VKH disease eyes in each stage at different follow-up time points. In the studied eyes, both pre- and posttreatment, there were statistically significant differences in BCVA and SFCT between the two stages of VKH disease. However, statistically significant variations in the grading of AC cells, the grading of AC flare, SRF height, and ICGA score were observed only before treatment. Specifically, compared with the eyes with late-stage VKH disease, the eyes with early-stage VKH disease had better BCVA (0.62 vs. 0.72, *p* = 0.015), lower AC cell grade (0.65 vs. 2.43, *p* < 0.001) and AC flare grade (0.78 vs. 1.94, *p* < 0.001), greater SFCT (621.87 vs. 445.62, *p* < 0.001) and SRF height (486.18 vs. 245.63, *p* < 0.001), and higher ICGA score (13.10 vs. 8.63, *p* < 0.001).

**Table 3:** Characteristics and comparison between the two different stages of VKH disease eyes at various follow-up times.

Characteristics	Early-stage			Late-stage			*p*-value		
	At baseline	At 2 months	At 6 months	At baseline	At 2 months	At 6 months	*p*_1_	*p*_2_	*p*_3_
BCVA (LogMAR)	0.62 ± 0.26	0.20 ± 0.13	0.08 ± 0.06	0.72 ± 0.24	0.29 ± 0.17	0.16 ± 0.06	0.015^*^	0.002^*^	< 0.001^*^
IOP (mmHg)	14.34 ± 1.06	15.11 ± 2.15	15.03 ± 4.55	14.65 ± 1.10	14.83 ± 3.02	13.97 ± 5.71	0.869	0.801	0.606
AC cells grade (+)	0.65 ± 0.15	0.11 ± 0.09	0	2.43 ± 1.25	0.35 ± 0.54	0.04 ± 0.61	< 0.001^*^	0.213	0.913
AC flare grade (+)	0.78 ± 0.21	0.09 ± 0.81	0	1.94 ± 1.53	0.22 ± 0.13	0.02 ± 0.06	< 0.001^*^	0.409	0.801
SFCT (µm)	621.87 ± 100.22	341.12 ± 86.30	303.62 ± 57.95	445.62 ± 116.81	254.97 ± 79.90	209.96 ± 62.95	< 0.001^*^	< 0.001^*^	< 0.001^*^
SRF height (µm)	486.18 ± 58.01	11.43 ± 8.63	0	245.63 ± 177.29	10.01 ± 9.05	0	< 0.001^*^	0.740	–
FA score	5.01 ± 2.31	1.05 ± 0.75	0.04 ± 0.15	3.98 ± 1.23	0.97 ± 0.61	0.09 ± 0.21	0.413	0.865	0.911
ICGA score	13.10 ± 2.83	4.03 ± 2.17	0.61 ± 0.91	8.63 ± 3.66	2.21 ± 0.94	0.64 ± 2.12	< 0.001^*^	0.613	0.982

Data are presented as mean ± SD (range). *p*-values for differences in variables between the early- and late-stage groups are based on a *t*-test. *p*_1_-, *p*_2_-, and *p*_3_-values indicate the differences between the two groups at baseline, 2 months, and 6 months, respectively.

*BCVA*, best-corrected visual acuity; *LogMAR*, logarithm of the minimum angle of resolution; *IOP*, intraocular pressure; *AC*, anterior chamber; *SFCT*, subfoveal choroidal thickness; *SRF*, subretinal fluid; *FA*, fluorescein angiography; *ICGA*, indocyanine green angiography.

^*^p < 0.05.

**Figure 2:**
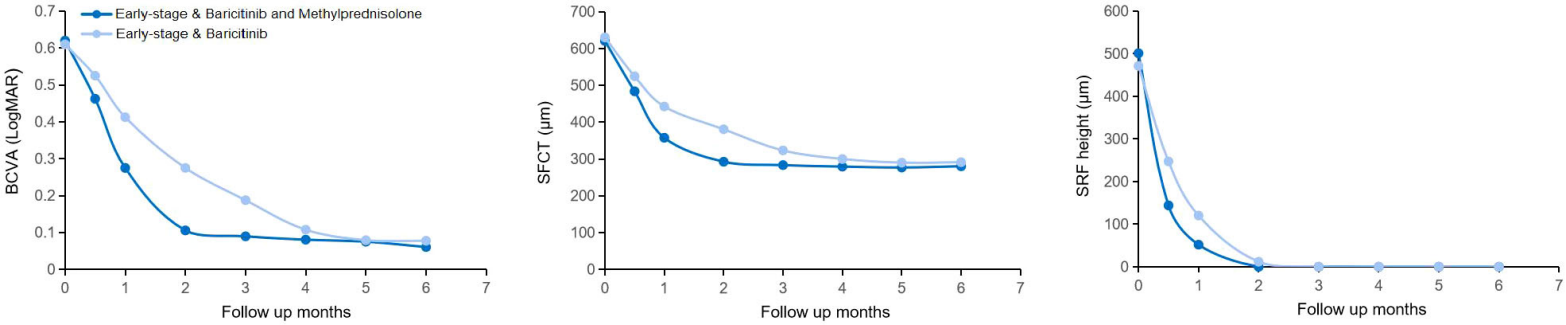
BCVA, SFCT, and SRF height changes during follow-up in two groups of early-stage VKH disease eyes with different dosing protocols. BCVA, best-corrected visual acuity; LogMAR, logarithm of the minimum angle of resolution; SFCT, subfoveal choroidal thickness; SRF, subretinal fluid.

**Figure 3:**
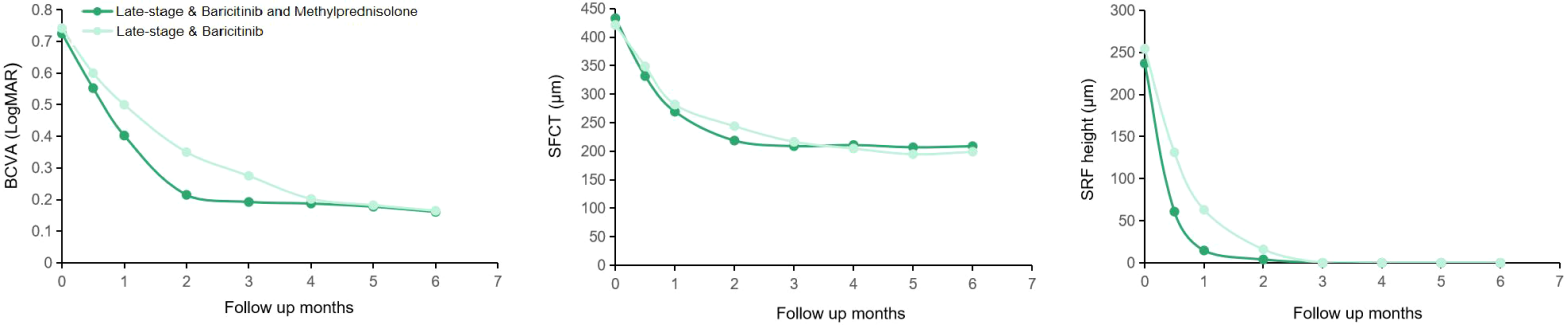
BCVA, SFCT, and SRF height changes during follow-up in two groups of late-stage VKH disease eyes with different dosing protocols. BCVA, best-corrected visual acuity; LogMAR, logarithm of the minimum angle of resolution; SFCT, subfoveal choroidal thickness; SRF, subretinal fluid.

**Figure 4:**
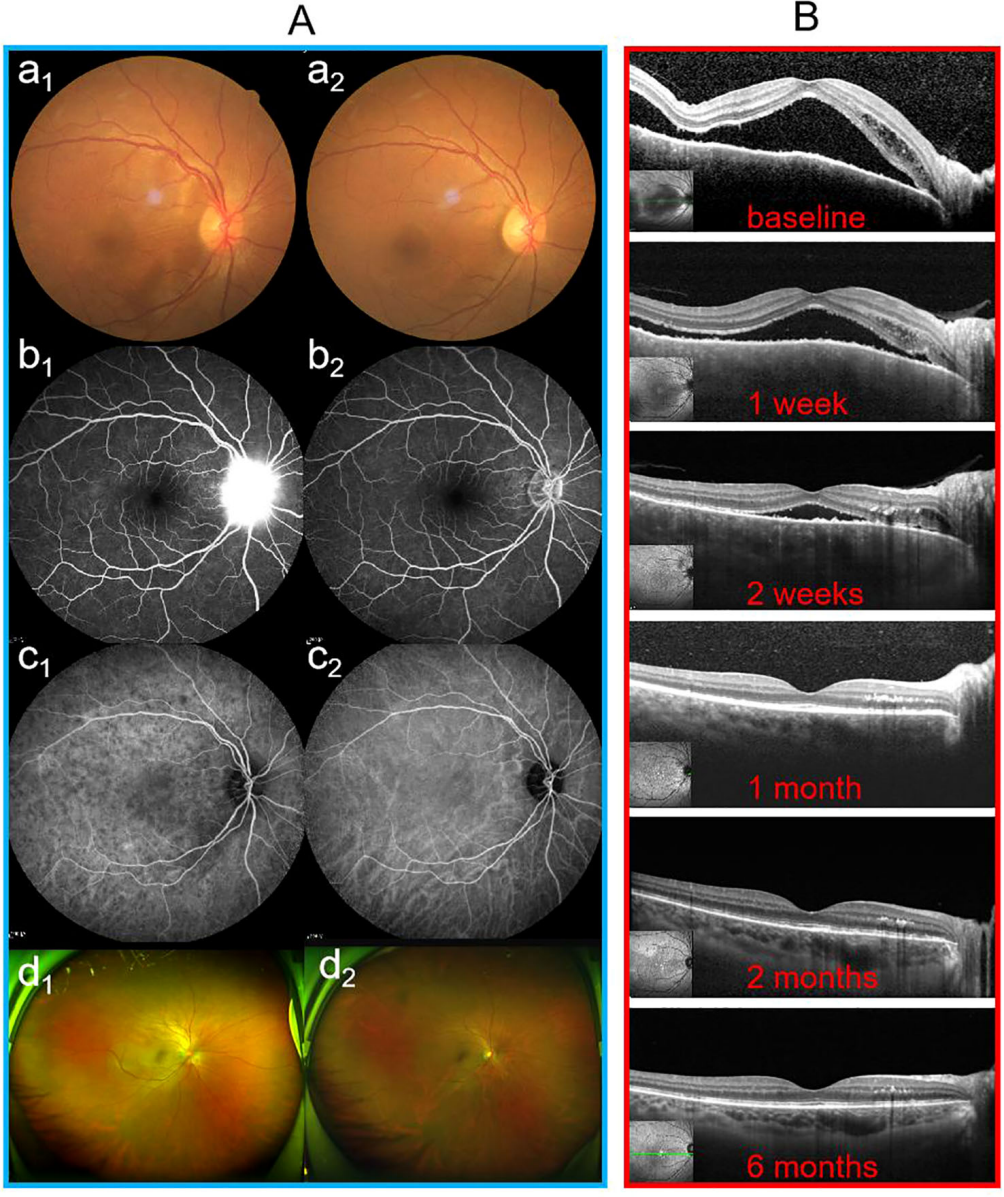
Follow-up findings for patient No. 1 with early-stage VKH disease who was treated with oral baricitinib monotherapy. **(A)** Multimodal imaging results at baseline (a_1_–d_1_) and at 6 months (a_2_–d_2_). **(B)** OCT images during the follow-up period. OCT, optical coherence tomography.

**Figure 5:**
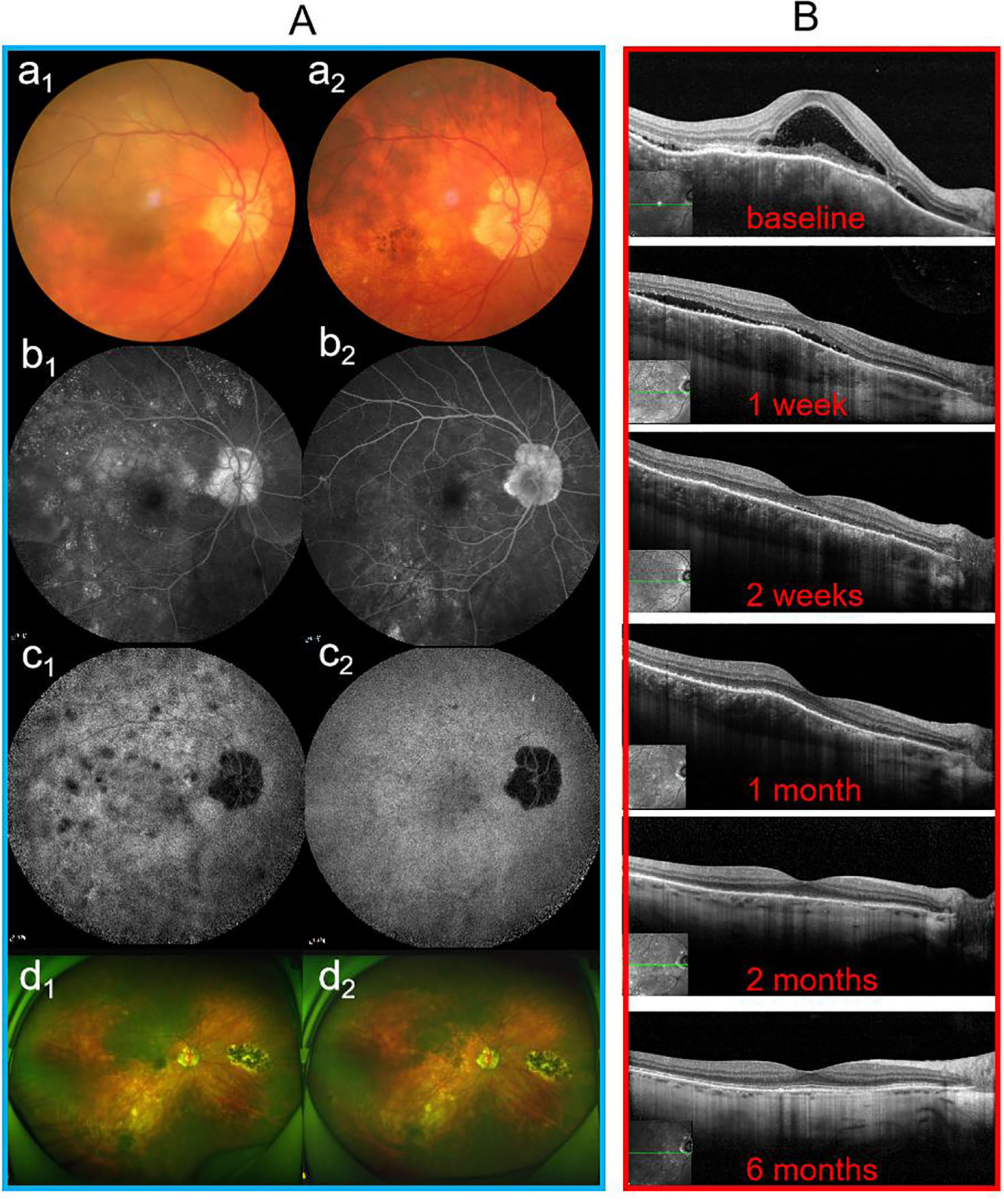
Follow-up findings for patient No. 22 with late-stage VKH disease who was treated with oral baricitinib monotherapy. **(A)** Multimodal imaging results at baseline (a_1_–d_1_) and at 6-month follow-up (a_2_–d_2_). **(B)** OCT images during the follow-up period. OCT, optical coherence tomography.

Following treatment, both groups achieved significant recovery, yet distinct clinical patterns were observed. Specifically, regarding visual outcomes, early-stage eyes achieved significantly better final BCVA compared to late-stage eyes, a trend maintained throughout the follow-up period. Anatomically, while SFCT decreased significantly in both groups, early-stage eyes exhibited a more profound reduction from baseline, consistent with the resolution of acute stromal edema. In terms of inflammatory control, vitreous haze (graded using the Nussenblatt scale) resolved rapidly in both groups, paralleling the clearance of anterior chamber cells; by the 6-month endpoint, no eyes in either group exhibited active vitritis or anterior chamber inflammation exceeding 0.5 +. As illustrated in **Figures 2**, **3**, this recovery involved rapid SRF absorption and visual gain, which were notably accelerated by the addition of methylprednisolone in the initial 2 months. Furthermore, multimodal imaging (**Figures 4**, **5**) confirmed the comprehensive resolution of optic disc hyperemia, choroidal folds, and angiographic leakage across both early- and late-stage eyes.

### Comprehensive analysis of pre- and posttreatment variables

A paired analysis of clinical parameters at baseline, 2 months, and 6 months is presented in **Table 4**. Overall, BCVA, AC cell/flare grades, SFCT, SRF height, and ICGA scores served as sensitive indicators of treatment response. Significant improvements in these parameters were observed as early as 2 months and were sustained at 6 months (*p* < 0.05 for all comparisons). In group B (monotherapy), the resolution of AC cells and flare was slower compared to group A at the 2-month time point, consistent with the delayed anti-inflammatory effect of JAK inhibitors compared to corticosteroids. However, by 6 months, complete resolution was achieved in group B as well. ICGA scores, representing choroidal inflammation, showed a significant reduction in all subgroups, correlating well with the decrease in SFCT.

**Table 4:** Further analysis of changes in variables for each group of patients with VKH disease over two follow-up periods.

Variables	Group A		Group B		Early-stage		Late-stage	
	*t*-value	*p*-value	*t*-value	*p*-value	*t*-value	*p*-value	*t*-value	*p*-value
BCVA (LogMAR)								
2 months vs. baseline	− 17.521	< 0.001^*^	− 18.280	< 0.001^*^	− 14.683	< 0.001^*^	− 20.663	< 0.001^*^
6 months vs. baseline	− 18.873	< 0.001^*^	− 18.112	< 0.001^*^	− 17.945	< 0.001^*^	− 19.105	< 0.001^*^
IOP (mmHg)								
2 months vs. baseline	0.568	0.588	− 0.778	0.462	0.231	0.824	0.168	0.981
6 months vs. baseline	− 0.651	0.497	0.690	0.492	0.476	0.593	− 0.688	0.493
AC cells grade (+)								
2 months vs. baseline	− 3.762	0.008^*^	− 1.528	0.170	− 1.113	0.324	− 5.643	< 0.001^*^
6 months vs. baseline	− 4.950	0.005^*^	− 4.101	0.007^*^	− 1.134	0.301	− 5.810	< 0.001^*^
AC flare grade (+)								
2 months vs. baseline	− 3.556	0.010^*^	− 1.426	0.197	− 0.981	0.365	− 5.334	< 0.001^*^
6 months vs. baseline	− 4.180	0.007^*^	− 4.345	0.006^*^	− 1.211	0.313	− 5.784	< 0.001^*^
SFCT (µm)								
2 months vs. baseline	− 19.509	< 0.001^*^	− 16.623	< 0.001^*^	− 23.322	< 0.001^*^	− 15.892	< 0.001^*^
6 months vs. baseline	− 23.989	< 0.001^*^	− 17.381	< 0.001^*^	− 28.295	< 0.001^*^	− 15.849	< 0.001^*^
SRF height (µm)								
2 months vs. baseline	− 27.906	< 0.001^*^	− 18.768	< 0.001^*^	− 29.012	< 0.001^*^	− 11.521	< 0.001^*^
6 months vs. baseline	− 28.014	< 0.001^*^	− 27.146	< 0.001^*^	− 29.156	< 0.001^*^	− 16.721	< 0.001^*^
FA score								
2 months vs. baseline	− 2.669	0.048^*^	− 1.809	0.106	− 2.506	0.053	− 2.192	0.062
6 months vs. baseline	− 3.089	0.020^*^	− 2.944	0.048^*^	− 3.260	0.016^*^	− 3.008	0.021^*^
ICGA score								
2 months vs. baseline	− 8.652	< 0.001^*^	− 6.348	0.002^*^	− 9.162	< 0.001^*^	− 6.864	0.004^*^
6 months vs. baseline	− 9.011	< 0.001^*^	− 9.211	< 0.001^*^	− 10.213	< 0.001^*^	− 8.309	< 0.001^*^
Total VFQ-25 score								
2 months vs. baseline	5.774	< 0.001^*^	6.377	< 0.001^*^	–	–	–	–
6 months vs. baseline	5.658	< 0.001^*^	6.012	< 0.001^*^				
Total SF-36 score								
2 months vs. baseline	1.528	0.171	1.537	0.168	–	–	–	–
6 months vs. baseline	1.610	0.175	1.540	0.165				

*t*- and *p*-values show differences of variables between the two follow-up time periods, which are based on a related-sample *t*-test. Values at 2 months vs. baseline indicate the differences between 2 months and baseline; values at 6 months vs. baseline indicate the differences between 6 months and baseline.

*BCVA*, best-corrected visual acuity; *LogMAR*, logarithm of the minimum angle of resolution; *IOP*, intraocular pressure; *AC*, anterior chamber; *SFCT*, subfoveal choroidal thickness; *SRF*, subretinal fluid; *FA*, fluorescein angiography; *ICGA*, indocyanine green angiography; *VFQ-25*, 25-item version of the Visual Function Questionnaire; *SF-36*, 36-item Short-Form Health Survey Questionnaire.

^*^p < 0.05.

## Discussion

To the best of our knowledge, this prospective study is the first to demonstrate the efficacy and safety of the JAK inhibitor baricitinib in the treatment of VKH disease. Our findings from this cohort, which represent the largest series of VKH patients treated with a JAK inhibitor to date, indicate that oral baricitinib effectively improved overall visual function, whether used as monotherapy or in combination with corticosteroids. This improvement was evidenced by significant gains in BCVA, a reduction in SFCT, complete absorption of SRF, and resolution of anterior chamber and fundus inflammation. Notably, while the addition of methylprednisolone in the initial 2 months accelerated the resolution of intraocular inflammation, the final 6-month outcomes were comparable between the combination and monotherapy groups. This suggests that while adjuvant corticosteroids may hasten early recovery, baricitinib alone is sufficient to maintain long-term remission.

The rationale for utilizing baricitinib in VKH disease is rooted in the specific immunopathogenesis of the condition and the limitations of current standard care. Conventionally, VKH is managed with high-dose systemic corticosteroids or immunosuppressive agents (36–38). However, long-term corticosteroid use is associated with severe systemic morbidity (e.g., hypertension, hyperglycemia) and ocular complications such as cataracts and glaucoma (21, 39). Furthermore, conventional immunosuppressants often exhibit a slow onset of action and variable efficacy (12, 40). Mechanistically, VKH disease is driven by an abnormal Th1 and Th17 immune response, with cytokines such as IFN-γ and IL-6 acting as key mediators (41, 42). These cytokines signal through the JAK-STAT pathway to activate downstream inflammatory cascades (21). Genetic studies have identified JAK1 polymorphisms as susceptibility loci for VKH in Han Chinese populations (43), and JAK2 upregulation is implicated in inflammatory cell migration in EAU (44). Baricitinib, as a selective inhibitor of JAK1 and JAK2, directly targets these distinct pathways (17). By inhibiting the phosphorylation of STAT proteins, baricitinib blocks the differentiation of pathogenic Th1 and Th17 cells and suppresses the “cytokine storm” characteristic of active VKH, providing a targeted therapeutic approach superior to broad-spectrum immunosuppression (45). We have summarized JAK inhibitors tested in clinical studies and basic trials for NIU in **Supplementary Table 2** (20, 23, 24, 46–51).

As a targeted small-molecule inhibitor, baricitinib possesses pharmacological characteristics distinct from biologic agents. Its small molecular mass and water solubility may facilitate penetration across physiological barriers, while the oral formulation improves patient compliance and convenience, making it suitable for long-term maintenance. Additionally, small molecules are generally not immunogenic, reducing the risk of antidrug antibody formation (52).

Our study also provides novel insights into the prognosis of early- vs. late-stage VKH disease. While baricitinib effectively controlled inflammation in both groups, a distinct difference in visual prognosis was observed. Early-stage patients achieved significantly better final BCVA compared to late-stage patients. This disparity highlights that while JAK inhibitors can arrest active inflammation and resolve subretinal fluid in chronic recurrent cases, they cannot reverse permanent photoreceptor damage or “sunset glow fundus” changes accumulated over time. Therefore, we emphasize the critical window of opportunity: initiating potent targeted therapy such as baricitinib in the early phase is paramount to preserving optimal visual function.

Regarding safety, baricitinib demonstrated a favorable profile in our cohort. According to product labeling, common adverse effects of JAK inhibitors include hyperlipidemia, cytopenia, and infections such as herpes zoster (17). In our study, no patients experienced severe adverse events, anemia, or malignancy. Specifically regarding herpes zoster, a known risk with JAK inhibitors, no cases were observed during follow-up. This may be attributed to the strict exclusion of high-risk patients and the relatively young and generally healthy demographic of patients with VKH compared with the rheumatoid arthritis population (53). Furthermore, consistent with the steroid-sparing nature of the protocol, no significant cataract progression or elevation of intraocular pressure was observed, addressing a major concern associated with conventional corticosteroid therapy.

Our study has several limitations. First, the single-center, nonrandomized design and the absence of a control group (e.g., standard corticosteroids or adalimumab) limit comparative efficacy conclusions and introduce potential selection bias. Second, while the 6–18-month follow-up captured acute outcomes, it may be insufficient to assess long-term safety and late recurrences. Third, the lack of aqueous humor drug level monitoring precluded direct confirmation of ocular penetration. Finally, the modest sample size restricted to a Chinese population may limit generalizability to other ethnicities. Future multicenter RCTs are needed, ideally comparing JAK inhibitors head-to-head against anti-TNF agents, to definitively establish their therapeutic positioning.

## Conclusions

In conclusion, we have discovered novel therapeutic alternatives for VKH disease in an effort to minimize or replace the use of corticosteroids, immunosuppressive agents, and other biologics. In the current study, the small-molecule agent baricitinib exhibited excellent effectiveness and safety in the treatment of VKH disease. During follow-up, this treatment was shown to control intraocular inflammation in patients with VKH disease, considerably enhance visual acuity, and was not associated with recurrence or severe adverse drug reactions. In the future, however, additional prospective studies with larger samples and longer durations, as well as mechanistic research in animal studies, are required.

## Data Availability

The original contributions presented in the study are included in the article/**Supplementary Material**. Further inquiries can be directed to the corresponding authors.
