# Supplementary material for: Novel application for the JAK inhibitor baricitinib in the treatment of Vogt–Koyanagi–Harada disease: a prospective cohort study
Source: Front Immunol. 2026 Mar 17;17:1736522. doi: 10.3389/fimmu.2026.1736522 (PMC13036186; doi:10.3389/fimmu.2026.1736522)
Supplement: Supplementary file 1 [file Table1.docx]

**Supplementary Table 1. Detailed demographic and clinical characteristics of all VKH patients.**

| **Patient no.** | **Gender** | **Age (years)** | **Disease categorization**  **/prior medication history)** | **Systemic features and extraocular manifestations of VKH** | **Ocular manifestations of VKH** | | **Ocular complications and ADR of baricitinib** | **Total follow-up (months)** |
| --- | --- | --- | --- | --- | --- | --- | --- | --- |
|  |  |  |  |  | **Anterior segment features** | **Posterior segment features** |  |  |
| 1* | M | 50 | OU Early-stage/  None | Headache, tinnitus;  Hypertension | OU AC cells 1/2+ | OU vitreous haze 1/2+, exudative retinal detachment, disc edema, increased choroidal thickness; optic disc hyperflfluorescence，macular edema，capillary leakage on FA； early stromal vessel hyperflfluorescence, dark dots, and optic disc hyperflfluorescence on ICGA | NA | 16 |
| 2* | F | 38 | OU Early-stage/  None | Meningismus | OU AC cells 1+, flare 1+ | OU exudative retinal detachment, disc edema, increased choroidal thickness；optic disc hyperflfluorescence，ｍacular edema and capillary leakage on FA；early stromal vessel hyperflfluorescence, dark dots, and optic disc hyperflfluorescence on ICGA | NA | 7 |
| 3* | M | 25 | OU Early-stage/  None | NA | OU AC cells 1/2+ | OU vitreous haze 1+, exudative retinal detachment,  disc edema, increased choroidal thickness; optic disc hyperflfluorescence，ｍacular edema，capillary leakage on FA； early stromal vessel hyperflfluorescence, choroidal vasculitis, dark dots, and optic disc hyperflfluorescence on ICGA | NA | 9 |
| 4* | M | 29 | OU Early-stage/  None | Hypertension | OU AC cells 1/2+, flare 1/2+ | OD vitreous haze 3+, and OS vitreous haze 1+, OU exudative retinal detachment, disc edema, increased choroidal thickness; optic disc hyperflfluorescence, ｍacular edema, capillary leakage on FA；early stromal vessel hyperflfluorescence, choroidal vasculitis, dark dots，and　optic disc hyperflfluorescence on ICGA | NA | 8 |
| 5* | F | 47 | OU Early-stage/  None | Headache, dysacusis | OU no AC cells/flare/KPs | OU exudative retinal detachment, disc edema, increased choroidal thickness；optic disc hyperflfluorescence，ｍacular edema and capillary leakage on FA；early stromal vessel hyperflfluorescence, dark dots, and optic disc hyperflfluorescence on ICGA | NA | 10 |
| 6* | F | 61 | OU Early-stage/  None | Headache | OD no AC cells/flare/KPs; OS AC cells 1/2+, flare 1/2+ | OU exudative retinal detachment, disc edema, increased choroidal thickness；optic disc hyperflfluorescence，ｍacular edema，and capillary leakage on FA；early stromal vessel hyperfluorescence, dark dots, and optic disc hyperflfluorescence on ICGA | OS complicated cataract | 12 |
| 7* | M | 37 | OU Early-stage/  None | NA | OU no AC cells/flare/KPs | OU exudative retinal detachment, disc edema, increased choroidal thickness；optic disc hyperflfluorescence，ｍacular edema，and capillary leakage on FA；early stromal vessel hyperfluorescence, dark dots, and optic disc hyperflfluorescence on ICGA | NA | 14 |
| 8* | M | 30 | OU Early-stage/  None | Tinnitus | OU no AC cells/flare/KPs | OU vitreous haze 2+, exudative retinal detachment, disc edema, increased choroidal thickness；optic disc hyperflfluorescence，ｍacular edema，capillary leakage，and ｐinpoint leaks on FA；early stromal vessel hyperfluorescence，choroidal vasculitis, dark dots, and optic disc hyperflfluorescence on ICGA | NA | 8 |
| 9* | F | 26 | OU Early-stage/  None | NA | OU AC cells 1/2+, flare 1+ | OD multifocal active choroiditis, OU disc edema, increased choroidal thickness；optic disc hyperflfluorescence, capillary leakage, pinpoint leaks, and subretinal pooling on FA; early stromal vessel hyperfluorescence, choroidal vasculitis, dark dots, and optic disc hyperflfluorescence on ICGA | NA | 11 |
| 10* | F | 59 | OU Early-stage/  None | Headache; Hypertension | OU AC cells 1+, flare 2+ | OU vitreous haze 1/2+, exudative retinal detachment, disc edema, increased choroidal thickness; optic disc hyperflfluorescence，macular edema，capillary leakage on FA； early stromal vessel hyperflfluorescence, dark dots, and optic disc hyperflfluorescence on ICGA | NA | 8 |
| 11* | F | 32 | OU Early-stage/  None | NA | OU no AC cells/flare/KPs | OU multifocal active choroiditis, disc edema, increased choroidal thickness；optic disc hyperflfluorescence, capillary leakage, pinpoint leaks, and subretinal pooling on FA; early stromal vessel hyperfluorescence, choroidal vasculitis, dark dots, and optic disc hyperflfluorescence on ICGA | OU intraocular hypertension | 12 |
| 12* | F | 58 | OU Early-stage/  None | Headache, tinnitus;  Hypertension | OD AC cells 1/2+, flare 1+, mutton fat KP; OS AC cells 1+, flare 1+ | OU vitreous haze 1/2+, exudative retinal detachment, disc edema, increased choroidal thickness；optic disc hyperflfluorescence，ｍacular edema，capillary leakage，and ｐinpoint leaks on FA；early stromal vessel hyperfluorescence，choroidal vasculitis, dark dots, and optic disc hyperflfluorescence on ICGA | NA | 6 |
| 11 | M | 54 | OU Early-stage/  None | NA | OU AC cells 1+, flare 2+ | OD multifocal active choroiditis, OU disc edema, increased choroidal thickness；optic disc hyperflfluorescence, capillary leakage, pinpoint leaks, and subretinal pooling on FA; early stromal vessel hyperfluorescence, choroidal vasculitis, dark dots, and optic disc hyperflfluorescence on ICGA | OD intraocular hypertension | 10 |
| 12 | F | 32 | OU Early-stage/  None | NA | OU no AC cells/flare/KPs | OU vitreous haze 3+, exudative retinal detachment, disc edema, increased choroidal thickness; optic disc hyperflfluorescence，macular edema，capillary leakage on FA； early stromal vessel hyperflfluorescence, dark dots, and optic disc hyperflfluorescence on ICGA | NA | 18 |
| 13 | F | 65 | OU Early-stage/  None | Tinnitus;  Diabetes mellitus | OU no AC cells/flare/KPs | OU multifocal active choroiditis, disc edema, increased choroidal thickness；optic disc hyperflfluorescence, capillary leakage, pinpoint leaks, and subretinal pooling on FA; early stromal vessel hyperfluorescence, choroidal vasculitis, dark dots, and optic disc hyperflfluorescence on ICGA | NA | 6 |
| 14 | F | 33 | OU Early-stage/  None | Headache | OD AC cells 1+, flare 1+, mutton fat KP; OS AC cells 1+, flare 1+ | OU exudative retinal detachment, disc edema, increased choroidal thickness；optic disc hyperflfluorescence，ｍacular edema，and capillary leakage on FA；early stromal vessel hyperfluorescence, dark dots, and optic disc hyperflfluorescence on ICGA | NA | 6 |
| 15 | M | 40 | OU Early-stage/  None | Meningismus | OU no AC cells/flare/KPs | OU vitreous haze 1/2+, exudative retinal detachment,  disc edema, increased choroidal thickness; optic disc hyperflfluorescence，ｍacular edema，capillary leakage on FA； early stromal vessel hyperflfluorescence, choroidal vasculitis, dark dots, and optic disc hyperflfluorescence on ICGA | OD complicated cataract | 11 |
| 16 | F | 27 | OU Early-stage/  None | Headache | OU no AC cells/flare/KPs | OD vitreous haze 1+, and OS vitreous haze 1/2+, OU exudative retinal detachment, disc edema, increased choroidal thickness; optic disc hyperflfluorescence, ｍacular edema, capillary leakage on FA；early stromal vessel hyperflfluorescence, choroidal vasculitis, dark dots，and　optic disc hyperflfluorescence on ICGA | NA | 9 |
| 17 | M | 30 | OU Early-stage/  None | NA | OU AC cells 1+, flare 2+ | OU exudative retinal detachment, disc edema, increased choroidal thickness；optic disc hyperflfluorescence，ｍacular edema and capillary leakage on FA；early stromal vessel hyperflfluorescence, dark dots, and optic disc hyperflfluorescence on ICGA | NA | 8 |
| 18 | F | 43 | OU Early-stage/  None | NA | OU no AC cells/flare/KPs | OU exudative retinal detachment, disc edema, increased choroidal thickness；optic disc hyperflfluorescence，ｍacular edema，and capillary leakage on FA；early stromal vessel hyperfluorescence, dark dots, and optic disc hyperflfluorescence on ICGA | NA | 17 |
| 19 | F | 39 | OD Early-stage  OS Late-stage/  None | Headache, tinnitus | OD no AC cells/flare/KPs；OS AC cells 1+, flare 1/2+ stellate KP | OU exudative retinal detachment, disc edema, increased choroidal thickness；optic disc hyperflfluorescence，ｍacular edema and capillary leakage on FA；OD sunset glow fundu, and OU early stromal vessel hyperflfluorescence, dark dots, and optic disc hyperflfluorescence on ICGA | NA | 9 |
| 20 | F | 58 | OU Early-stage/  None | Diabetes mellitus | OU AC cells 1+, flare 2+ | OU exudative retinal detachment, disc edema, increased choroidal thickness；optic disc hyperflfluorescence，ｍacular edema，and capillary leakage on FA；early stromal vessel hyperfluorescence, dark dots, and optic disc hyperflfluorescence on ICGA | NA | 7 |
| 21 | M | 57 | OU Early-stage/  None | Headache | OU no AC cells/flare/KPs | OU vitreous haze 1/2+, exudative retinal detachment, disc edema, increased choroidal thickness；optic disc hyperflfluorescence，ｍacular edema，capillary leakage，and ｐinpoint leaks on FA；early stromal vessel hyperfluorescence，choroidal vasculitis, dark dots, and optic disc hyperflfluorescence on ICGA | NA | 12 |
| 22* | M | 44 | OU Late-stage/  Corticosteroids+  Methotrexate | NA | OU AC cells 2+, flare 1+ | OU exudative retinal detachment, disc edema, increased choroidal thickness, sunset glow fundus, Dalen Fuchs–like nodules; capillary leakage on FA；choroidal vasculitis on ICGA | NA | 8 |
| 23* | F | 24 | OU Late-stage/  None | NA | OU AC cells 3+, flare 3+,  iris posterior synechiae | OU vitreous haze 3+, exudative retinal detachment, disc edema, increased choroidal thickness, sunset glow fundus; optic disc hyperflfluorescence, ｍacular edema and capillary leakage on FA; stromal vessel　hyperfluorescence, dark dots，and optic disc hyperflfluorescence on ICGA | OD subretinal fibrosis | 10 |
| 24* | F | 32 | OU Late-stage/  Corticosteroid | NA | OD AC cells 1+, flare 1+ | OU vitreous haze 2+; window defects fluorescence, capillary leakage on FA；dark dots, and choroidal vasculitis on ICGA | NA | 13 |
| 25* | M | 62 | OD Late-stage  OS Early-stage/  Corticosteroid | NA | OU AC cells 2+, flare 2+,OD fine KP, iris posterior synechiae | OD vitreous haze 1+, OU exudative retinal detachment, increased choroidal thickness, OD sunset glow fundus; OU optic disc hyperflfluorescence, macular edema，capillary leakage，pinpoint leaks , and subretinal pooling on FA；stromal vessel hyperfluorescence，choroidal vasculitis，dark dots, and optic disc hyperflfluorescence on ICGA | NA | 18 |
| 26* | F | 46 | OU Late-stage/  None | NA | OU AC cells 3+, flare 3+,  iris posterior synechiae | OU vitreous haze 4+, exudative retinal detachment, disc edema, increased choroidal thickness, sunset glow fundus; optic disc hyperflfluorescence, ｍacular edema and capillary leakage on FA; stromal vessel　hyperfluorescence, dark dots，and optic disc hyperflfluorescence on ICGA | NA | 9 |
| 27* | F | 51 | OU Late-stage/  Corticosteroid | Poliosis | OU AC cells 3+, flare 3+, fine KP | OU exudative retinal detachment, disc edema, increased choroidal thickness, sunset glow fundus; optic disc hyperflfluorescence，ｍacular edema, capillary leakage，pinpoint leaks, and subretinal pooling on FA；stromal vessel hyperfluorescence，choroidal vasculitis, dark dots, and optic disc hyperflfluorescence on ICGA | NA | 8 |
| 28* | M | 36 | OU Late-stage/  None | NA | OS AC cells 1+, flare 1/2+ | OU vitreous haze 1+, multifocal active choroiditis; optic disc hyperflfluorescence，window defects fluorescence, and capillary leakage on FA；stromal vessel hyperfluorescence and optic disc hyperflfluorescence on ICGA | NA | 6 |
| 29* | F | 29 | OU Late-stage/  None | NA | OU AC cells 2+, flare 3+ | OU vitreous haze 2+; window defects fluorescence, capillary leakage on FA；dark dots, and choroidal vasculitis on ICGA | NA | 15 |
| 30* | F | 62 | OU Late-stage/  None | Poliosis;  Diabetes mellitus | OU AC cells 4+ | OU vitreous haze 1/2+, multifocal active choroiditis; optic disc hyperflfluorescence, window defects fluorescence, and capillary leakage on FA；stromal vessel hyperfluorescence and optic disc hyperflfluorescence on ICGA | OU intraocular hypertension | 7 |
| 31* | F | 51 | OU Late-stage/  Corticosteroids | NA | OU AC cells 3+, flare 2+ | OU vitreous haze 3+, exudative retinal detachment, disc edema, increased choroidal thickness, sunset glow fundus; optic disc hyperflfluorescence, ｍacular edema and capillary leakage on FA; stromal vessel　hyperfluorescence, dark dots，and optic disc hyperflfluorescence on ICGA | NA | 6 |
| 32 | F | 61 | OU Late-stage/  Corticosteroids | NA | OS AC cells 1/2+, flare 1/2+ | OU vitreous haze 1/2+, sunset glow fundus, Dalen Fuchs–like nodules; optic disc hyperflfluorescence, capillary leakage, pinpoint leaks， and subretinal pooling on FA; stromal vessel hyperfluorescence, dark dots, choroidal vasculitis and optic disc hyperflfluorescence on ICGA | NA | 6 |
| 33 | M | 39 | OU Late-stage/  None | Hypertension | OU AC cells 3+, flare 3+, fine KP | OU exudative retinal detachment, disc edema, increased choroidal thickness, sunset glow fundus, Dalen Fuchs–like nodules; capillary leakage on FA；choroidal vasculitis on ICGA | NA | 6 |
| 34 | M | 43 | OU Late-stage/  Corticosteroids+ Cyclosporine A | NA | OD AC cells 1+, flare 1/2+；OS AC cells 3+, flare 2+ | OU vitreous haze 1+, exudative retinal detachment, increased choroidal thickness, sunset glow fundus; optic disc hyperflfluorescence, macular edema，capillary leakage，pinpoint leaks , and subretinal pooling on FA；stromal vessel hyperfluorescence，choroidal vasculitis，dark dots, and optic disc hyperflfluorescence　on ICGA | NA | 8 |
| 35 | M | 58 | OU Late-stage/  None | Hypertension; Diabetes mellitus | OD AC cells 4+, flare 3+; OS AC cells 3+, flare 3+, mutton fat KP, iris posterior synechiae | OU vitreous haze 1/2+, sunset glow fundus, Dalen Fuchs–like nodules; optic disc hyperflfluorescence, capillary leakage, pinpoint leaks， and subretinal pooling on FA; stromal vessel hyperfluorescence, dark dots, choroidal vasculitis and optic disc hyperflfluorescence on ICGA | OS subretinal fibrosis | 12 |
| 36 | F | 47 | OU Late-stage/  corticosteroids | Alopecia, poliosis | OD AC cells 3+, flare4+ | OU vitreous haze 3+, exudative retinal detachment, disc edema, increased choroidal thickness, sunset glow fundus; optic disc hyperflfluorescence, ｍacular edema and capillary leakage on FA; stromal vessel　hyperfluorescence, dark dots，and optic disc hyperflfluorescence on ICGA | NA | 15 |
| 37 | M | 41 | OU Late-stage/  corticosteroids | NA | OU AC cells 3+, flare 3+,  iris posterior synechiae | OU exudative retinal detachment, disc edema, increased choroidal thickness, sunset glow fundus; optic disc hyperflfluorescence，ｍacular edema, capillary leakage，pinpoint leaks, and subretinal pooling on FA；stromal vessel hyperfluorescence，choroidal vasculitis, dark dots, and optic disc hyperflfluorescence on ICGA | OU complicated cataract | 18 |
| 38 | F | 28 | OU Late-stage/  None | Alopecia | OU AC cells 3+, flare 3+, fine KP | OU exudative retinal detachment, disc edema, increased choroidal thickness, sunset glow fundus, Dalen Fuchs–like nodules; capillary leakage on FA；choroidal vasculitis on ICGA | NA | 9 |
| Total | M/F=  16/22 | 43.10±12.32 | Early-stage/Late-stage=  44 eyes/32 eyes | / | / | / | / | 10.2 ± 5.8 |

*Ｆ, female; Ｍ, male; OU, oculus uterque;* *OD, oculus dexter; OS, oculus sinister; ADR, adverse drug reaction; AC, anterior chamber; FA,fluorescein angiography; ICGA,indocyanine green angiography. * represents patients on early 2-month shock therapy using oral methylprednisolone with baricitinib.*

**Supplementary Table 2. JAK inhibitors tested in clinical trials for NIU.**

| **Jakinibs** | **JAK specificity** | **Reported articals** | | | | | | | |
| --- | --- | --- | --- | --- | --- | --- | --- | --- | --- |
|  |  | **author** | **type** | **subjects** | **Dose of Jakinibs** | **Side effects and toxicity of Jakinibs** | **Associated**  **other drugs while on Jakinibs** | **Follow-up** | **Clinical remission** |
| Tofacitinib | JAK3/JAK1>JAK2, TYK2 | Paley et al.[46]  (2018) | Case report | A refractory NIU adult | 11 mg/day | None | MTX | 3 months | 100%(1 case) |
|  |  | Bauermann et al.[47]  (2019) | Case report | A JIA-uveitis associated with refractory ME adult | 10 mg/day | None | MTX 2.5 mg/d | 14 months | 100%(1 case) |
|  |  | Bing et al.[48]  (2020) | Original article | EAU B10.A mice model | 25 mg/kg/day | None | None | 14 days | / |
|  |  | Dutta et al.[24]  (2020) | Case report | A VKH disease adult | 10 mg/day | None | corticosteroid | 1 month | 100%(1 case) |
|  |  | Miserocchi et al.[49] (2020) | Case series | A refractory JIA-uveitis adult | 10 mg/day | None | None | 7 months | 100%(1 case) |
|  |  | Vitale et al.[23]  (2024) | Original article | A NIU adult | 10 mg/day | None | prednison | 8 months | 100%(1 case) |
| Baricitinib | JAK1/JAK2 | Miserocchi et al.[49] (2020) | Case series | Three refractory JIA-uveitis adults | 4 mg/day | None | MTX 15 mg/wk | 4-13 months | 100%(3 cases) |
|  |  | Kaneko et al.[50]  (2022) | Case report | A panuveitis associated with seronegative RA adult | 8 mg/day | None | None | 2 years and 7 months | 100%(1 case) |
|  |  | Ramanan et al.[20]  (2021) | Study protocol | JIA-uveitis or chronic ANA-positive uveitis children | 4 mg/d (≥6 to <18 years) and 2 mg/d (<6 years) | / | DMARDs, low-dose corticosteroids, and/or NSAIDs | 2-5years | / |
|  |  | Vitale et al.[23]  (2024) | Original article | Two NIU adults and children | 2-4 mg/day | None | prednison | 3-17 months | 100%(4 case) |
| Upadacitinib | JAK1 | Vitale et al.[23]  (2024) | Original article | Seven NIU adults | 15 mg/day | None | immunosuppressant | 3-20 months | 100%(7 case) |
| Filgotinib | JAK1 | Erdağ et al.[51]  (2021) | Original article | EIU rats model | 3 mg/kg/day | None | None | 10 days | / |

*JAK: Janus kinase; TYK: tyrosine kinase; NIU, non-infectious uveitis; MTX, methotrexate; JIA, juvenile idiopathic arthritis; ME, macular edema; EAU, experimental autoimmune uveitis; IFN-γ, [interferon-gamma](http://www.baidu.com/link?url=ZUNSW1-H6JaEzo9hed44HtV89tVp5f2261fjPjkwVj6XpEt3mHa2h8j1XNy6yC46qyQ3BCw0xPkDVYV4tnvywhUvXGOopS1BYTHQF8fCH7E59aIT0e1v2w4UbhdgwuFLQvKAos2yjgdOrGtYlknlAY3wV4KCeZFY4eJ8c4_yzTz5nBYlEj_RV80IW8uj6xTu78_iM2KU8BTgM098ncuXFKqu_2mLcAod6p6mboTqiWKwD9E-iqL5iyKEYtoJSnpjhV_Pq33DZ41HSFDvPVTz_FwBBDiM5j3IsvB5jnvMKtveoowESy5SkVhxfO5YupeJejH0LNm67xcrsvmVkvCkhJAZSNrfzZkPENsUQF90YzHU2rMHANkdCxgaA6WUTY-60CnUv82Zze_oRt6bOVGe__); VKH, Vogt-Koyanagi-Harada disease; RA, rheumatoid factor; ANA, antinuclear antibody; DMARDs, disease-modifying antirheumatic drugs; NSAIDs, nonsteroidal anti-inflammatory drugs; EIU, endotoxin-induced uveitis.*
